# Asthma and Obstructive Sleep Apnea Overlap in a Sample of Older American Indian Adults: The Strong Heart Study

**DOI:** 10.3390/jcm13185492

**Published:** 2024-09-17

**Authors:** Huimin Wu, Dorothy A. Rhoades, Jessica A. Reese, Kellie R. Jones

**Affiliations:** 1 Pulmonary, Critical Care and Sleep Medicine Section, Department of Medicine, University of Oklahoma Health Sciences Center, Oklahoma City, OK 73104, USA; kellie-jones@ouhsc.edu; 2 Stephenson Cancer Center, University of Oklahoma Health Sciences Center, Oklahoma City, OK 73104, USA; dorothy-rhoades@ouhsc.edu; 3 Department of Biostatistics and Epidemiology, Center for American Indian Health Research, University of Oklahoma Health Sciences Center, Oklahoma City, OK 73104, USA; jessica-reese@ouhsc.edu; 4 Pulmonary, Critical Care and Sleep Medicine, Oklahoma City VA Health Care System, Oklahoma City, OK 73104, USA

**Keywords:** asthma, obstructive sleep apnea, American Indians, Strong Heart Study

## Abstract

**Study Objectives:** Our study aimed to investigate the association between asthma and obstructive sleep apnea (OSA) in American Indian communities, a historically underrepresented population in clinical research with a high prevalence of asthma and OSA risk factors like smoking and obesity. **Methods:** This cross-sectional study used data retrieved from the Strong Heart Study cohort. Participants who attended both the Asthma Sub-study and the Sleep Heart Health Study around the same time were compared for active asthma diagnosis, OSA diagnosis, and potential risk factors for asthma and OSA. The association between asthma and OSA was then evaluated. **Results:** Among the 2480 participants who attended the Strong Heart Study Phase III exam, 123 participated in both the Asthma Sub-study and the Sleep Heart Health Study. Of these, 13 were diagnosed with OSA, with 4 having moderate to severe OSA. There was no statistically significant difference in OSA prevalence between the active asthma group and the non-active asthma group (former asthma or no asthma) (9.6% vs. 12.5%, *p* = 0.63). Additionally, body mass index did not differ significantly between participants with both active asthma and OSA and those without active asthma, OSA, or both. OSA diagnosis was significantly associated with male sex (Odds Ratio [OR] 9.2 [1.85–45.87], *p* = 0.007) and body mass index (OR 1.1 [1.02–1.26], *p* = 0.016) but not with age or a diagnosis of active asthma. **Conclusions:** In this American Indian cohort, no significant difference in OSA prevalence was observed between participants with and without active asthma, contradicting previous studies. Further research is needed to explore the underlying reasons for this discrepancy.

## 1. Introduction

Asthma is characterized by variable airflow obstruction, leading to symptoms such as dyspnea and wheezing [1]. It is one of the most prevalent chronic respiratory diseases worldwide, affecting approximately 300 million individuals globally, with a reported prevalence of asthma symptoms around 6–7% in adults [1]. After decades of increasing prevalence, asthma rates now appear to be plateauing. The condition is frequently associated with comorbidities that can complicate its management and worsen symptoms. Common comorbidities include allergic rhinitis, gastroesophageal reflux disease, obesity, anxiety, depression, chronic obstructive pulmonary disease, cardiovascular disease, and obstructive sleep apnea (OSA), among others [1].

OSA is characterized by recurrent episodes of partial or complete collapse of the upper airway during sleep, leading to reduced (hypopnea) or absent (apnea) airflow lasting for at least 10 seconds, often accompanied by either cortical arousal or a fall in blood oxygen saturation^2^. OSA affects approximately 25% of adults in the U.S., and its prevalence increased by about 30% between 1990 and 2010 [2]. The prevalence of OSA increases with age. In the U.S., the prevalence of OSA is approximately 43.2% in men and 27.8% in women among non-Hispanic white individuals aged 50 to 70 years [2]. The estimated prevalence of OSA among African American adults aged 50 to 80 years was 53.6%. OSA has been shown to be associated with various cardiovascular complications, including hypertension, arrhythmias, heart failure, coronary artery disease, stroke, pulmonary hypertension, metabolic syndrome, diabetes, and an increased risk of cardiovascular mortality [3]. Observational studies also revealed that patients with OSA have an increased risk of asthma, and OSA is associated with worse asthma severity, increased frequency of exacerbation, and poor quality of life.

In the past twenty years, with increased awareness of OSA, more attention has been directed toward investigating the relationship between asthma and OSA. Available data suggest a bidirectional relationship between asthma and OSA, with each condition potentially influencing the other, likely due to shared risk factors such as airway and systemic inflammation, chronic intermittent hypoxia, lung remodeling, and airway dysfunction [4,5,6]. However, clinical study results have not always been consistent. For example, a meta-analysis indicated that asthma had no significant effect on OSA [7]. Additionally, data from a large sleep health study revealed that the severity of OSA in non-asthmatic patients was greater than in asthmatic patients [8]. This discrepancy is thought to be due to differences between the cohorts: the non-asthmatic control group was predominantly male and older, both of which are known risk factors for severe OSA, while the asthmatic cohort was predominantly female and younger.

Most studies examining the relationship between OSA and asthma have focused on Caucasian and African American populations [5,9,10,11]. However, American Indian adults have a 50% higher likelihood of obesity compared to Caucasians, which is a major risk factor for OSA [12]. In fact, one study found that American Indians had 1.7 times higher odds of moderate to severe OSA compared to Caucasians [13]. Additionally, cigarette smoking, a common risk factor for respiratory diseases, is more prevalent among American Indians than other ethnic groups [14]. As a result, the potential associations between OSA and asthma in American Indian patients may differ from those in other ethnic groups. Although some studies have included American Indian adults with asthma [15,16,17,18,19] or OSA [13,20,21,22], no study has yet investigated the relationship between these two conditions in this population. The overlap between asthma and OSA has gained increasing attention over the past twenty years; however, there is a lack of data specifically within the American Indian population to investigate the relationship between these conditions. To address this gap, we conducted a study using data from a large cohort of American Indians to explore the association between asthma and OSA. Analyzing these data now presents a valuable opportunity to investigate these conditions in a population that has been historically underrepresented in such studies. Our goal is to provide preliminary insights that can serve as a foundation for future research in this area. Our hypothesis is that active asthma is associated with both the diagnosis and severity of OSA. 

## 2. Methods

**Figure 1:**
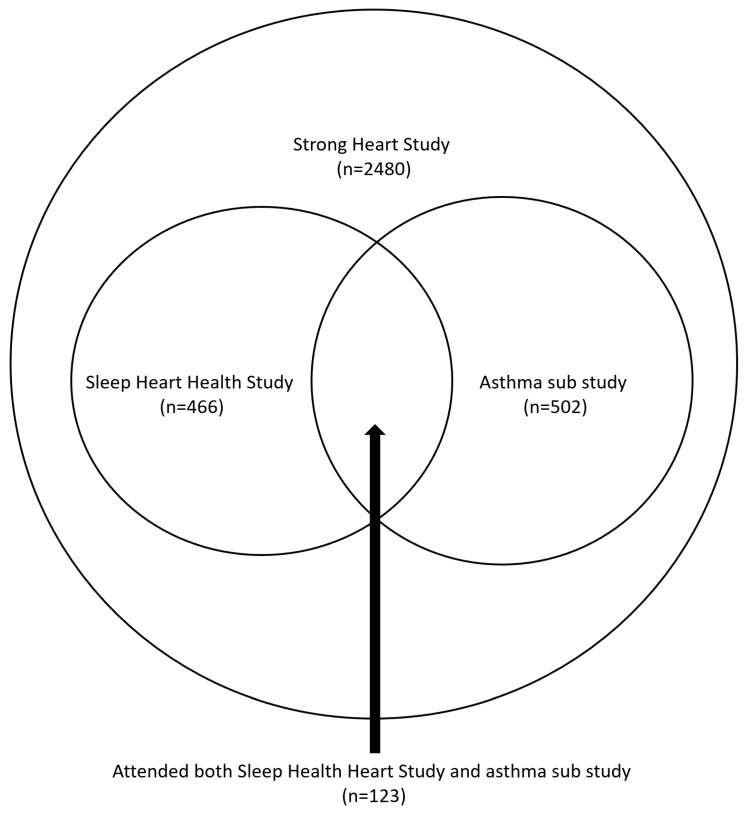
Participant enrollment.

The Strong Heart Study (SHS) is a multi-center, longitudinal cohort study of cardiovascular disease in American Indian communities, supported by the National Heart, Lung, and Blood Institute since 1 October 1988 [23]. It is the largest epidemiological study of American Indians ever undertaken. The SHS has completed six clinical examinations of the original and family cohorts (Phase I: 1989–1991; Phase II: 1993–1995; Phase III: 1998–1999; Phase IV: 2000–2005; Phase V: 2005–2010; and Phase VI: 2014–2018). The Phase VII clinical examination is currently ongoing. Between 1995 and 1998, a sub-study of the cohort, the Sleep Heart Health Study (SHHS), was conducted [24]. In addition, a separate asthma sub-study was conducted between 1998 and 1999 [15]. Some participants in the SHS took part in both sub-studies, providing an opportunity to investigate the interplay between asthma and OSA in this population. The present study utilized information collected from the SHS, asthma sub-study, and the SHHS to establish the eligibility criteria, assess asthma and OSA severity, and evaluate potential risk factors (Figure 1). Informed consent was obtained from participants for the SHS, asthma sub-study, and the SHHS. This study was approved by the SHS steering committee, and permission for the publication of this manuscript was granted by the institutional review boards of all participating tribes.

### 2.1. Strong Heart Study (SHS)

Detailed methods for the SHS sample are described elsewhere [25]. In brief, the SHS used a standardized methodology and was designed to estimate cardiovascular disease mortality and morbidity rates, as well as the prevalence of known and suspected cardiovascular disease risk factors in American Indians. Investigators recruited adults aged 45–74 years from 12 tribal communities in central Arizona, southwestern Oklahoma, North Dakota, and South Dakota beginning in 1988. Approximately 4500 tribal members underwent clinical examinations at that time (Phase I). Personal interviews were conducted to assess family history, diet, alcohol and tobacco consumption, physical activity, degree of acculturation, and socioeconomic status. A comprehensive physical examination was performed, and laboratory measurements were taken, including glucose, insulin, fasting lipids, glycated hemoglobin, serum and urine creatinine, and urinary albumin. The SHS Phase III exam was conducted between 1998 and 1999 and included an update on personal habits and medical history, an assessment of alcohol and tobacco consumption, measures of height, weight, and blood pressure, an evaluation of peripheral vascular disease, a 12-lead resting electrocardiogram, ultrasound assessment of arteries, and laboratory measurements. Basic participant characteristics in our study, including age, sex, smoking history, height, and weight (for body mass index [BMI] calculation), were collected from the SHS Phase III exam database. 

### 2.2. Asthma Sub-Study

The Asthma Sub-study was conducted simultaneously with the SHS Phase III exam from 1998 to 1999. Participants were recruited from the same 12 tribal communities as the SHS. In-person interviews were conducted on the same date and at the same location as the SHS Phase III exam. A sample of participants aged 50 years or older completed a standardized respiratory questionnaire, performed spirometry, and underwent allergen skin testing. Those participants who were enrolled in the asthma sub-study had a positive response to any of the following screening questions: (1) Self-reported physician-diagnosed asthma; (2) Had attacks of wheezing with shortness of breath; (3) Shortness of breath with wheezing at night; and/or (4) Use of asthma inhalers in the last 12 months. Those who had congestive heart failure or a history of more than 20 pack-year smoking were excluded from the asthma sub-study to reduce confounding by a competing diagnosis. Among those who were included, participants were asked three additional questions: 1. “At any time during the last 12 months, have you had wheezing or whistling in your chest?” 2. “Have you ever had asthma?” and “If “yes,” do you still have asthma?”. Participants were defined as having physician-diagnosed active asthma or probable active asthma if they answered “yes” to the question “At any time during the last 12 months, have you had wheezing or whistling in your chest?” or answered “yes” to both “Have you ever had asthma?” and “If “yes,” do you still have asthma?” [15]. The remaining participants were included in no active asthma group. In addition, spirometry measurements were collected from participants who were willing and able to cooperate with the test. 

### 2.3. Sleep Heart Health Study (SHHS) 

The SHHS is a multi-center cohort study aimed at determining the cardiovascular and other consequences of sleep-disordered breathing [24]. Its objective was to investigate whether sleep-related breathing was associated with an increased risk of coronary heart disease, stroke, all-cause mortality, and hypertension. From 1995 to 1998, participants were recruited from multiple existing epidemiological studies in which data on cardiovascular risk factors had been previously collected, including the SHS, the Framingham Offspring Cohort, the Atherosclerosis Risk in Communities study, the Cardiovascular Health Study, studies of respiratory disease in Tucson, and hypertension in New York. For this present analysis, we used data from SHS participants who attended the SHHS. Inclusion criteria were as follows: (1) Age 40 years or older; (2) No history of treatment for sleep apnea; (3) No tracheostomy; and (4) No current home oxygen therapy [24]. Participants were recruited through targeted mailings, telephone calls, and during clinic visits for SHS phase III examinations. Participants were scheduled for a home polysomnogram as soon as possible after recruitment. Data collected during the home examination included the following: seated blood pressure, weight, neck circumference, health interview (questions pertaining to prevalent cardiovascular and respiratory disease and smoking), current medications, quality of life questionnaire, modified Epworth sleepiness scale, morning sleep quality survey, and home polysomnogram. The Compumedics P Series System (Abbotsford, Victoria, Australia) was selected as the primary data acquisition tool. Data were reviewed initially at each clinical site and then transmitted to a central polysomnography reading center that was established to provide uniform, standardized scoring and quality assessment determinations for all polysomnographic data. Final scoring was performed manually on an epoch-by-epoch basis. Home polysomnogram examination results, including the quality and duration of the study and the severity of sleep apnea, were collected for this study. OSA was defined according to the presence of related symptoms and the frequency of respiratory events during sleep recorded in the SHHS. The severity of OSA was classified using the respiratory event index (REI). Mild OSA was defined as an REI of 5–15 events per hour, moderate OSA as an REI of 15–30 events per hour, and severe OSA as an REI greater than 30 events per hour. 

### 2.4. Data Request Process

The investigators of this project completed the online SHS Data Request form, along with the SHS Data Distribution Agreement form, and provided a study protocol detailing the research objectives and intended uses of the data. For both SHS and asthma sub-study data, the investigators specified the required variables based on the Phase III data dictionary and the Phase III asthma sub-study data dictionary [23]. For SHHS data, the variables were selected based on information provided on the SHHS website [26].

### 2.5. Statistical Analysis

The participants were categorized based on four criteria: (1) participation in the SHHS, (2) participation in the asthma sub-study, (3) diagnosis of OSA, and (4) diagnosis of physician-diagnosed active asthma or probable active asthma. Differences between the 2 group comparisons were assessed using a non-paired t-test for normally distributed variables and a Kruskal–Wallis rank-sum test for non-normally distributed data. Proportions between groups were tested using χ^2^ analysis. When the expected cell count for any group was less than 5, Fisher’s exact test was used to assess statistical significance. A *p*-value < 0.05 was considered statistically significant. Multiple logistic regression analysis was performed to determine the association between the diagnosis of OSA and the diagnosis of asthma, adjusted for age, sex, and BMI, which are common covariates that may affect the diagnosis of OSA. The interactions between the diagnosis of asthma and age, sex, and BMI were also evaluated. All analyses were carried out using STATA statistical software (STATA 18, StataCorp, College Station, TX, USA).

## 3. Results

Out of the 2480 participants who attended the SHS Phase III exam, 466 participated in the SHHS, 502 participated in the asthma sub-study, and 123 participated in both the SHHS and the asthma sub-study (Table 1, Figure 1). There was no difference in age between the group that attended both the SHHS and the asthma sub-study and those who did not participate in either study (mean [±SD] 62.7 ± 0.7 vs. 63.7 ± 0.2, *p* = 0.15). There was no significant difference in sex between the groups either (female 58.5% vs. 61.6%, *p* = 0.50). Slightly more women than men participated in each of the studies, with the majority of participants being in their 60s. Those who attended both the SHHS and the asthma sub-study had a significantly higher BMI than those who did not (mean [±SD] 32.1 ± 6.5 vs. 30.8 ± 6.2, *p* = 0.02). There was no difference in smoking history between participants who attended both the asthma sub-study and the SHHS and those who did not attend either study (65.9% vs. 66.1%, *p* = 0.99). 

The overall information regarding home sleep studies is presented in Table 2. The quality of all the sleep studies was acceptable for data analysis. As shown in Table 3, out of the 123 participants who attended both the SHHS and the asthma sub-study, 13 had OSA (REI ≥ 5), of whom 4 had moderate to severe OSA (REI ≥ 15). There was no statistically significant difference in OSA diagnosis between the active asthma group and the non-active asthma group (9.6% vs. 12.5%, *p* = 0.63). Among those diagnosed with OSA, there was no significant difference in OSA severity between active asthma and no active asthma groups (REI mean [±SD] 8.8 ± 4.0 vs. 16.7 ± 9.2, *p* = 0.06). The BMI did not differ in participants who had both active asthma and OSA and those who did not (mean [±SD] 35.5 ± 7.56 vs. 31.8 ± 6.34, *p* = 0.11). 

The forced expiratory volume in 1 second as a percentage of predicted (FEV1 %pred) was lower in men with active asthma compared to those without (mean [±SD] 76.9 ± 3.4% vs. 95.5 ± 3.3%, *p* = 0.001). There was no significant difference in FEV1 %pred between women with active asthma and those without (mean [±SD] 74.3 ± 4.3% vs. 77.7 ± 6.9%, *p* = 0.676). Additionally, there was no significant difference in FEV1 %pred between participants with and without OSA (see Supplemental Table S1).

Multiple logistic regression showed that diagnosis of OSA was associated with male sex (Odds Ratio [OR] 9.2 [1.85–45.87], *p* = 0.007) and BMI (OR 1.1 [1.02–1.26], *p* = 0.016), but not with age or diagnosis of asthma (see Supplemental Table S2). 

## 4. Discussion

The primary objective of this cross-sectional study was to investigate the association between asthma and OSA in American Indian communities. This population has a high prevalence of obesity, which is a major risk factor for OSA, and smoking, which has been linked to airway inflammation and respiratory diseases. Therefore, this study aimed to fill the knowledge gap in this area by examining the relationship between asthma and OSA in American Indian participants for the first time. Despite the high prevalence of both conditions in this population, our study found no significant association between the prevalence of asthma and OSA.

The airway and systemic inflammation caused by asthma may destabilize the control mechanisms of peripheral and central breathing as well as the upper airway, potentially leading to OSA [5]. Long-term use of inhaled corticosteroids for asthma can cause anatomical changes in the pharyngeal airway, increasing the likelihood of upper-airway collapse during sleep and contributing to an increased risk of OSA. Conversely, OSA and its risk factors may also lead to suboptimal overall asthma control [4]. Chronic intermittent hypoxia induced by OSA has been demonstrated to alter the airway inflammatory profile, resulting in lung remodeling, airway dysfunction, and a reduced response to inhaled corticosteroid therapy. OSA treatment has been shown to improve asthma symptoms, improved peak expiratory flow rates, and quality of life [27]. Current asthma clinical guidelines include untreated OSA as a potential contributor to poor asthma control [28].

Several studies have explored the relationship between asthma and OSA in non-American Indian populations. A study enrolled 22 patients with severe, unstable asthma who were receiving long-term oral corticosteroid therapy. It found that all but one participant had OSA, with a mean BMI of around 29 kg/m² [10]. Another study from Canada enrolled 78 participants with varying levels of asthma severity and a control group without asthma [29]. OSA was found in 50% of participants with severe asthma, 23% with moderate asthma, and 12% in the control group. A multi-center study enrolled 401 participants to determine the prevalence of OSA risk among patients with asthma of different severity and normal controls [30]. Participants completed the Sleep Apnea Scale of the Sleep Disorder Questionnaire and clinical assessments. Participants with a higher asthma burden were found to have a higher prevalence of OSA symptoms. A population-based prospective epidemiologic study in Wisconsin examined the prospective relationship of asthma with incident OSA beginning in 1988 [31]. All participants attended overnight polysomnography studies at 4-year intervals. A total of 22 of 81 participants (27%) with asthma experienced incident OSA in the 4-year follow-up interval compared with 75 of 466 participants (16%) without asthma. 

Obesity is a common risk factor for both OSA and asthma, with obesity playing a major role in the development of OSA [5]. In addition, obesity is a significant risk factor for incident asthma and the severity of the condition. Previous studies suggested that obesity is a major risk factor linking asthma with OSA [5]. Thus, it is possible that the association between OSA and asthma could be influenced by higher BMI. American Indians have a high prevalence of obesity [32]. Our study found that participants who took part in both the SHHS and asthma sub-study had a higher BMI compared to the entire cohort, and higher BMI was associated with the diagnosis of OSA. However, we did not observe a significant difference in BMI between those who had both OSA and active asthma and those who did not.

Compared to previous studies that evaluated OSA in asthma patients with smaller or similar sample sizes [10,29,31], our study cohort had a lower prevalence of OSA, both in participants with and without active asthma. Additionally, contrary to other studies, our study did not reveal a higher prevalence of OSA in participants with active asthma compared to those without active asthma. There are several possible reasons for these differences. First, our cohort was not selected randomly but rather based on meeting specific inclusion and exclusion criteria in the respective groups being studied. A retrospective review was conducted, which combined separate sub-studies of OSA and asthma, each with different inclusion criteria. This method may have led to the exclusion of individuals with competing diagnoses, such as heart disease and chronic obstructive pulmonary disease. Second, the asthma research was carried out subsequent to the home polysomnogram rather than concurrently, potentially leading to bias. Third, race-specific factors, such as differences in mechanical stress from OSA and obesity or anatomical effects of long-term inhaled corticosteroid therapy, could not be assessed in our study. Finally, it has been previously and repeatedly demonstrated that the seemingly lower prevalence of other chronic diseases among American Indian populations has often been due to significant errors in data collection rather than a racially specific protective predisposition [33,34,35]. Larger, systematic studies of respiratory diseases among adult American Indian populations are needed.

## 5. Conclusions

Our study found that, among American Indian participants, there was no significant difference in the prevalence of OSA between those with and without active asthma. These exploratory data are contrary to findings from other studies that have evaluated the relationship between OSA and asthma. Further studies are needed to investigate the underlying causes of these differences.

## Figures and Tables

**Table 1:** Demographics of participants.

Participant Groups	Strong Heart Study (n = 2480)	Sleep Heart Health Study (n = 466)	Asthma Sub-Study (n = 502)	Attended both Sleep Health Heart Study and Asthma Sub-Study (n = 123)
Female sex, n (%)	1523 (61)	276 (59)	339 (68)	72 (59)
Age, yr, mean (range)	63 (51–84)	62 (51–84)	64 (52–83)	63 (52–80)
BMI, kg/m^2^, mean (SD)	30.8 (6.2)	30.7 (6.1)	31.2 (6.8)	32.1 (6.5)
Smoking, n (%)				
Never	810 (32.7)	159 (34.1)	154 (30.7)	42 (34.1)
Ever	845 (34.1)	177 (38.0)	175 (34.9)	43 (35.0)
Current	734 (29.6)	124 (26.6)	167 (33.3)	38 (30.9)
Missing data	91 (0.3)	6 (1.3)	6 (1.2)	0
Smoking pack-years, median (IQR) *	12 (4–29)	13 (4–35)	15 (5–37)	13 (5–38)
Lung function				
FEV1 %pred, mean (SD)			77 (28)	78 (25)
FEV1/FVC ratio, mean (SD)			0.75 (0.45)	0.76 (0.30)
REI, median (IQR)		0.3 (0–1.8)		0.3 (0–1.5)

* for participants who were ever or current smokers only. BMI: body mass index, SD: standard deviation, IQR: interquartile range, FEV1 %pred: forced expiratory volume in 1 second as a percentage of predicted, FEV1/FVC: forced expiratory volume in one second to forced vital capacity ratio.

**Table 2:** Overall home sleep study results for participants who attended both SHHS and asthma sub-study.

Metric	Values
Recording time, minutes, mean (SD)	438 (69)
Sleep time, minutes, mean (SD)	349 (73)
Sleep study quality grade, n (%)	
- Outstanding	26 (21)
- Excellent	32 (26)
- Very good	17 (14)
- Good	26 (21)
- Fair	22 (18)
REI, median (IQR)	0.3 (0–1.5)

SD: standard deviation; IQR: interquartile range; REI: respiratory event index.

**Table 3:** Characteristics and OSA distribution according to the asthma status for participants who attended both SHHS and asthma sub-study.

	Physician Diagnosed Active Asthma or Probable Active Asthma, n = 83	No Active Asthma, n = 40
Female sex, n (%)	51 (61)	21 (53)
Age, yr, mean (range)	63 (52–80)	63 (52–80)
BMI, kg/m^2^, mean (SD)	32.6 (6.8)	31.0 (5.7)
Smoking, n (%)		
- Never	26 (31)	16 (40)
- Ever	32 (39)	11 (28)
- Current	25 (30)	13 (33)
OSA severity, n (%)		
- No OSA	75 (90.4)	35 (87.5)
- Mild OSA	7 (8.4)	2 (5)
- Moderate OSA	1 (1.2)	2 (5)
- Severe OSA	0	1 (2.5)

SD: standard deviation.

## Data Availability

Strong Heart Study data are shared with researchers following Resource and Data Sharing Policies. Researchers can request data by following the instructions (https://strongheartstudy.org/Research/Study-Data-and-Study-Samples/Study-Data#391231915-data-and-summary-statistics-request-policy, accessed on 15 September 2024).

## Supplementary Materials

The following supporting information can be downloaded at: https://www.mdpi.com/article/10.3390/jcm13185492/s1, Table S1. The forced expiratory volume in 1 second as a percentage of predicted (FEV1 %pred) for participants who attended both SHHS and asthma sub-study. Table S2. Multivariable logistic regression analyses for predictors of obstructive sleep apnea odds ratios.

## Abbreviations

BMI	Body mass index
FEV1 %pred	Forced expiratory volume in 1 second as a percentage of predicted
FEV1/FVC	Forced expiratory volume in one second to forced vital capacity ratio
IQR	Interquartile range
OR	Odds Ratio
OSA	Obstructive sleep apnea
REI	Respiratory event index
SD	Standard deviation
SHHS	Sleep Heart Health Study
SHS	Strong Heart Study
