# Supplementary material for: Asthma and Obstructive Sleep Apnea Overlap in a Sample of Older American Indian Adults: The Strong Heart Study
Source: J Clin Med. 2024 Sep 17;13(18):5492. doi: 10.3390/jcm13185492 (PMC11432384; doi:10.3390/jcm13185492)
Supplement: Supplementary file 1 [file jcm-13-05492-s001.zip › jcm-3174961-supplementary.pdf]

Supplementary Material

Table S1. The forced expiratory volume in 1 second as a percentage of predicted (FEV1 %pred) for participants who attended both SHHS and asthma sub study

		active asthma or probable active asthma		P value	OSA		P value
		Yes	No		Yes	No	
FEV1 %pred, mean (SD)	Male	76.9±3.4	95.5±3.3	0.001	85.8±4.1	83.5±3.2	0.762
	Female	74.3±4.3	77.7±6.9	0.676	80.7±15.9	75.0±3.8	0.741

SD: standard deviation

Table S2. Multivariable logistic regression analyses for predictors of obstructive sleep apnea Odds Ratios

Predictors	Odds Ratio	95% Confidence Interval	P value
Sex	9.201	1.846 to 45.871	0.007
Age	1.069	0.982 to 1.163	0.123
BMI	1.134	1.023 to 1.256	0.016
Asthma	0.839	0.207 to 3.397	0.806

BMI: body mass index.
